# Modified Rotated Flap With Double-Component Releasing Incision and One-Sided Tunnel for Mandibular Gingival Recession: A Case Report

**DOI:** 10.1155/crid/6046186

**Published:** 2025-08-23

**Authors:** Ta Dong Quan, Nguyen Quan Pham, Van Nhan Vo

**Affiliations:** ^1^Department of Periodontics and Implantology, Nhan Tam Dental Maxillofacial Speciality Hospital, Ho Chi Minh City, Vietnam; ^2^Department of Prosthodontics, Faculty of Odonto-Stomatology, Hong Bang International University, Ho Chi Minh City, Vietnam; ^3^Department of Implantology, Faculty of Odonto-Stomatology, Hong Bang International University, Ho Chi Minh City, Vietnam

**Keywords:** case report, gingival recession, rotation, subperiosteal, surgical flaps, tissue transplantation

## Abstract

**Background:** Several techniques have been proposed to deal with gingival recession and gain positive effectiveness and clinically acceptable results. Nevertheless, most require a two-stage surgical procedure with several drawbacks. The present clinical report describes the treatment procedure and clinical outcomes of a modified rotated flap technique incorporating a double-component releasing incision and one-sided tunnel approach for the management of gingival recession.

**Methods:** A 32-year-old male nonsmoker presented with a recession of 6 mm at tooth 33 and 8 mm at tooth 43 after orthodontic treatment. An 8 × 12 × 1.5-mm-size connective tissue graft was harvested from the hard palate. A tunnel flap was created on the mesial side of the canine using split-thickness technique. A reduced incision was placed distal to the second premolar, consisting of a curved segment that followed the cervical contours of the canine and premolars, and concluded with a double-portion releasing incision. The graft was inserted into the tunnel, and the gingival flap was repositioned and secured to cover the exposed tooth surface.

**Results:** After the 6-month and 1-year follow-ups, the mean root coverage rate was 100% without pain and tooth sensitivity.

**Conclusions:** Although additional studies and long-term follow-up will be needed to evaluate the effectiveness of this procedure, this case report showed that this technique not only increases root coverage but also keratinized the mucosa width in only one surgical appointment.

**Summary**

This technique could be used for managing severe gingival recession combined with increasing the width of keratinized gingiva in only one surgical appointment.

• Why is this case new information?

◦ Presentating and describing of a modified rotated flap technique, incorporating a double-component releasing incision and one-sided tunneling, to manage advanced gingival recession and augment keratinized tissue in a single surgery.

◦ The laterally rotated flap combined with double portion incision improves flap stability and preserves keratinized gingiva.

◦ A full-thickness rotated flap is beneficial for thin phenotypes, preserving blood supply, and reducing the risk of flap necrosis.

• What are the keys to the successful management of this case?

◦ Ensuring secure graft stabilization and reducing flap tension are crucial for achieving full coverage of the exposed root surface.

• What are the primary limitations to success in this case?

◦ Further studies are required to verify the effectiveness and reliability of this technique.

◦ Creating a wide flap can cause pain for the patient due to increased tissue trauma.

## 1. Introduction

Gingival recessions are highly prevalent and often associated with hypersensitivity, the development of caries and noncarious cervical lesions on the exposed root surface and impaired esthetics [1]. This condition involves a reduction in gingival tissue thickness and periodontal attachment. As a result, it may cause aesthetic concerns, increase tooth sensitivity, and enhance carious lesion as well as periodontal disease [2]. The etiology of gingival recession includes mechanical factors, periodontal pathology, occlusal trauma, or parafunctional habits [3, 4]. Additionally, it can occur following orthodontic treatment, particularly in the lower anterior region [5]. This is more common in cases of thin biotype and thin buccal bone.

Over the recent decades, various surgical techniques have been developed to address this condition [6]. In cases with a shallow vestibule, achieving full root coverage often required two surgical stages. The first step was a free gingival graft, which is followed by a second procedure with a coronally advanced flaps after the site has fully healed [7]. This approach presents two major drawbacks: increased patient morbidity due to the need for a second surgery and suboptimal aesthetic outcomes due to differing appearance between the palatal donor site and the recipient site [8]. Especially in the anterior mandibular region, surgical procedures are frequently complicated by shallow vestibular depth and a deficiency of keratinized tissue. These conditions can result in increased flap tension, particularly due to the attachment of underlying structures following coronal tissue traction [9]. Similar techniques have already been described in the literature, such as the lateral closed tunnel technique [10] and the laterally rotated flap technique [11]. Referencing these established approaches provides important clinical context and supports the rationale for proposing a novel surgical modification. With a very limited number of randomized controlled trials, these techniques had several limitations such as high tension risk, technical demands, limited tissue advancement, and difficult in nonideal biotypes [6].

Therefore, this case report presents an improved methodology by incorporating a modified rotated flap with a double-component releasing incision and a one-sided tunnel, which combines root coverage and keratinized gingival graft for mandibular anterior teeth. It outlines the procedure, highlights the outcomes, and emphasizes the technique's effectiveness in addressing both functional and aesthetic challenges in periodontal therapy.

## 2. Materials and Methods

### 2.1. Case Presentation

**Figure 1:**
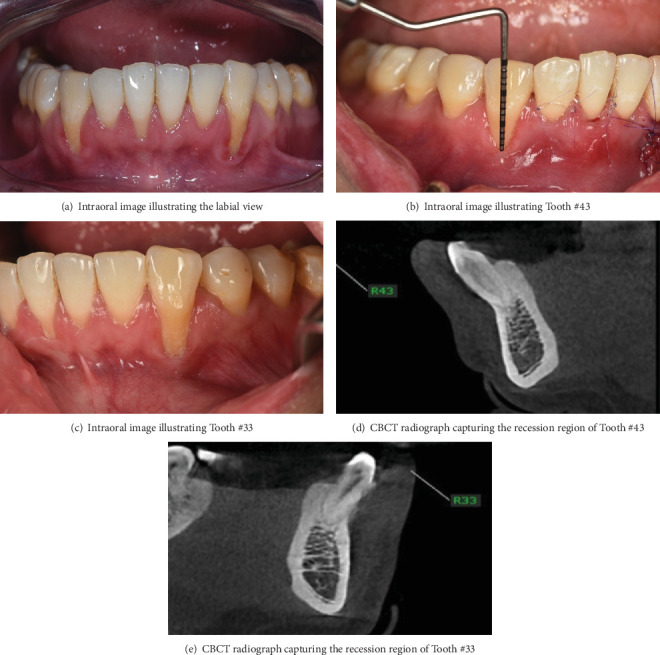
Preoperative evaluation: (a–e) A 32-year-old male exhibits gingival recession in the lower left and right canines.

A 32-year-old male nonsmoker with no systemic conditions, including diabetes or cardiovascular disease, presented with severe gingival recession, tooth sensitivity accompanied by noticeable marginal soft tissue inflammation and was referred to the Periodontal Department at Nhan Tam Dental Maxillofacial Speciality Hospital, Vietnam. A thorough clinical examination was conducted, which included a periodontal assessment and evaluation of the gingival biotype. The patient was examined and diagnosed with gingival recession on Teeth 33 and 43, classified as Cairo Class I (Figures 1a, 1b, and 1c) [12]. The patient had undergone comprehensive orthodontic treatment 2 years earlier. Marginal tissue recession was measured as the distance from the cementoenamel junction (CEJ) to the gingival margin. A recession of 6 mm at Tooth 33 and 8 mm at tooth 43, along with bleeding on probing, was observed. The width of keratinized tissue, the gingival thickness, and the probing depth were 1 mm each. Clinical attachment level, which was measured from the CEJ to the base of the alveolar bone, of Tooth 33 was 7 mm and that of Tooth 43 was 9 mm. Panoramic and cone-beam computed tomography (CBCT) assessments were performed. The sagittal cross-sectional view of the CBCT scan revealed the absence of buccal bone plate at the affected tooth (Figure 1d,e). This case report was prepared in accordance with the ethical principles outlined in the Declaration of Helsinki (1964) and its subsequent revisions. Written informed consent was obtained from the patient, including agreement to the proposed treatment plan and permission for the publication of clinical details and accompanying images. CARE guidelines were followed in this case study [13].

### 2.2. Presurgical Procedure

Dental scaling was performed prior to surgery, especially in the lower anterior region. After 2 weeks, gingival inflammation had decreased.

#### 2.2.1. 2.3. Surgical Procedures

Before flap preparation, a syringe of PrefGel (Straumann PrefGel 0.6 mL [24% EDTA, Basel, Switzerland]) was applied for 2 min to remove the smear layer and expose root surfaces. To obtain a connective tissue graft (CTG), an 8 × 12 × 1.5-mm-size graft was collected from the hard palate in the area from the first premolar to the first molar after de-epithelializing with high-speed diamond bur under abundant irrigation until a bleeding surface is visualized. Prior to use, Emdogain (Straumann Emdogain, Basel, Switzerland) which had been stored in a refrigerator at temperatures about 4°C was allowed to reach room temperature for 15 min. Emdogain was subsequently applied across the entire root surface, beginning at the most apical level of the root.

**Figure 2:**
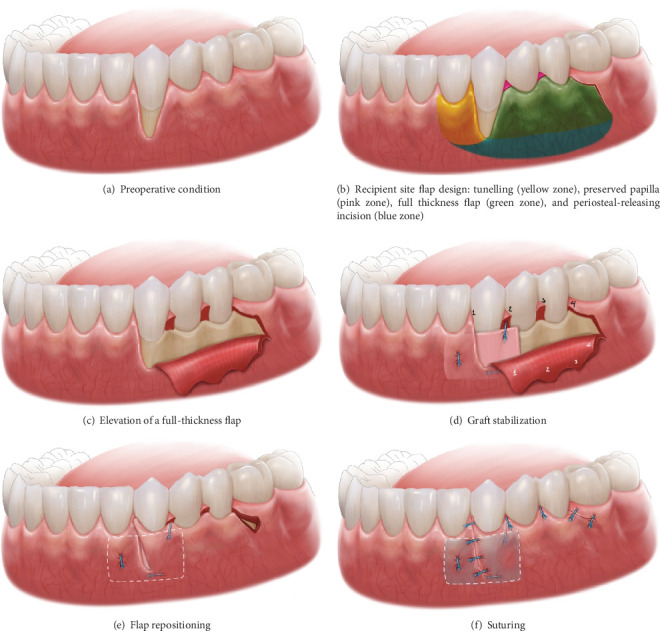
(a–f) Step-by-step illustration of the surgical procedure.

**Figure 3:**
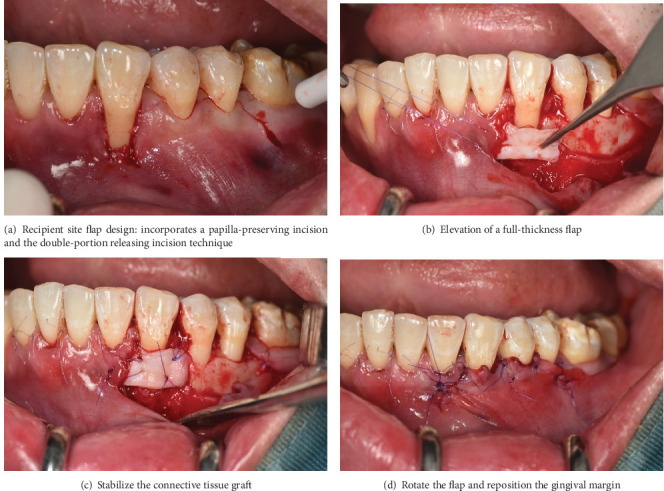
(a–d) Clinical images of the surgical procedure.

On the mesial side of the canine, where gingival recession was observed (Figures 2a and 3a), a split-thickness tunnel flap was created using a microsurgical tunneling instrument Hu-Friedy TKN1. On the distal side of the canine, a full-thickness flap was performed with a preserved papilla technique and a periosteal-releasing incision at the vestibular depth to reduce tension while maintaining full thickness in the central portion of the flap using a 15C blade. A double-portion incision was made distal to the second premolar to further reduce tension. This incision consisted of a curved segment following the gingival contour of the first molar, positioned approximately 2 mm from the gingival margin, along with a straight incision (Figure 2b).

The CTG graft was inserted into the tunnel through the sulcular access on the mesial side of the canine, with the epithelial portion positioned over the entire exposed root (Figure 2c,d). The graft sites were then secured with 6-0 PDS sutures (Figure 3b,c). After de-epithelialization of the interdental papillae, the gingival flap was rotated, repositioned, and sutured with 6-0 PDS to cover the entire connective tissue area (Figures 2e, 2f, and 3d). The same procedure was performed on the contralateral canine.

### 2.3. 2.4. Postoperative Care and Monitoring

**Figure 4:**
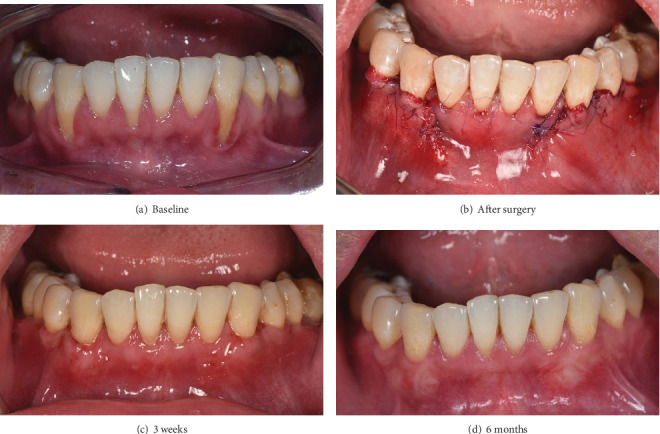
(a–d) Clinical images of postoperative follow-up.

**Figure 5:**
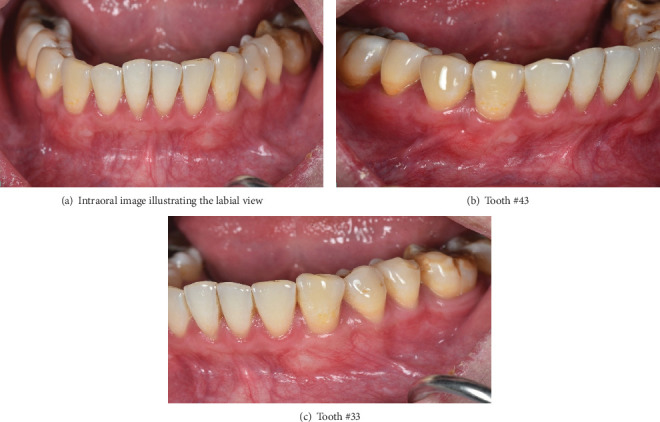
(a–c) The clinical photograph at 1 year postoperative.

**Figure 6:**
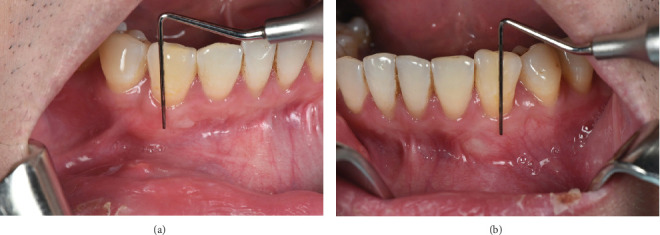
Measurement of keratinized mucosa width at (a) Tooth #43 and (b) Tooth #33 at 1 year postoperative.

The patient received the following postoperative instructions: avoid brushing the surgical area for 2 weeks, rinse with 0.12% chlorhexidine twice daily for 1 min, take 500 mg of paracetamol immediately after the procedure and 6 h later if pain persists, and use a soft toothbrush for a minimum of 3 months. The patient was closely monitored following surgery to ensure proper healing and optimal outcomes. If any abnormal swelling, bleeding, or infection was detected, the patient was informed to return for checkup immediately. At the 3-week and 1-year postoperative follow-ups, the patient returned to the periodontal department for evaluation (Figure 4). Clinical examination revealed that the free gingival margin remained stable at the initial position achieved after surgery. The width of keratinized tissue was approximately 4 mm and the gingival thickness was 1.2 mm. The patient reported no pain or tooth sensitivity. Gingival recession and bleeding on probing were absent. After 1 year, a complete root coverage was achieved at both canine sites (Figures 5 and 6).

## 3. Discussions

Various treatment methods have been introduced for cases of gingival recession in the lower anterior teeth region. This necessitates the evaluation of the effectiveness of various contemporary surgical methods to address this issue adequately. The average coverage of the root surfaces in the anterior lower jaw region was recorded at 95.7%, compared with 100% for the maxillary anterior [14]. The highest mean root coverage percentages were linked to the combination of laterally positioned flap with CTG at 91.2% and tunnel with CTG at 89.4%. In contrast, the standalone use of laterally positioned flap, coronally advanced flap with CTG, and free gingival graft yielded lower mean root coverage percentages at 79.1%, 78.9%, and 68.5%, respectively [15]. Harris' study reported the success rate of modified root coverage with CTG and coronally positioned flap to be 90.9% for cases when the gingival recession depth is less than 3 mm. However, the success rate exhibited a notable decrease to 68.4% for cases when the recession depth was equal to or exceeded 3 mm [16]. Yotnuengnit et al. established a strong correlation between root coverage outcomes and the ratio of graft tissue area to visible denuded area. Specifically, the results emphasized that achieving comprehensive root coverage required a minimum graft tissue area to visible denuded area ratio of 11:1 [17]. According to Han et al., the CTG exposed by around 1–2 mm exhibited no statistically significant difference in root coverage [18]. Nevertheless, ensuring the success rate of root coverage cannot be guaranteed when the graft is exposed by more than 1–2 mm. Consequently, with a large amount of gingival recession depth in this case report (more than 6 mm), it was mentioned to fully envelop the graft with a flap to enhance its viability and survival rate [15].

Currently, abundant evidence supported the effectiveness of tunnel grafting techniques in treating mild-to-moderate recession in the anterior mandibular region. The laterally closed tunnel technique, introduced by Sculean and Allen in 2018, had demonstrated reliable outcomes when applied to coverage procedures at the tooth root in the lower jaw area [10]. However, in cases of bilateral symmetrical recession, this technique presents significant challenges in reducing flap tension. In 2018, Agusto et al. applied the gingival pedicle combined with the split-thickness tunnel technique to address single deep recessions on mandibular incisors [19]. However, due to the thin biotype commonly found in the mandibular incisor region, utilizing the split-thickness tunnel technique can pose challenges or limitations in surgical execution. CTG in combination with a split-thickness tunnel flap has been shown to be effective in managing gingival recession defects, particularly in cases with thin biotype, root prominence, and inadequate keratinized tissue [20]. The tunnel technique preserves interdental papillae and maintains vascular supply, while the CTG augments tissue thickness and promotes long-term stability and root coverage. This approach minimizes flap trauma and improves esthetic integration, making it especially suitable for challenging anatomical sites [21].

In this case report, to ensure the success of grafting, a combination of one-sided tunnel with double-component releasing incision was used in a modified rotated flap approach to manage the thin biotype found in the mandibular incisor region. Despite the absence of buccal bone extending 6–8 mm apical to the CEJ, complete root coverage was achievable without bone augmentation when interdental clinical attachment is preserved. Studies have shown that even in cases of buccal dehiscence, ideal soft tissue outcomes are possible, provided the pedicle maintains blood supply and flap tension is minimized [22]. The aim of this technique was to gain at least 2 mm of keratinized mucosa width with adequate thickness; this can prevent future gingival recession [23, 24]. This modified technique can be suggested for treatment of gingival recession classified as Cairo Class I. The laterally rotated flaps combining with the tunneling graft were performed to create maximum coverage of gingival recession of the canines [5, 25]. Stabilization of the CTG enhanced the healing process after surgery [26]. Therefore, a complete root coverage implies a 100% coverage of the exposed root surface back to the CEJ, restoring the gingival margin to its original anatomic position [6]. Nevertheless, the double portion incision at the premolar region can reduce the tension of the full-thickness flap and avoid secondary healing. In fact, a tight suture was easily fixed on the cervical of the second premolar because of a longer incision line. In our previous study, only one portion incision was performed, which lead to the loss of keratinized tissue after surgery because of the tension of the flap [5].

In this study, Emdogain was applied to the graft site to enhance the success rate of the surgery because of severe recession and single surgical procedure. Clinical research had shown that the application of Emdogain gel resulted in a statistically significant increase in root coverage, gain in the clinical attachment level, and probing pocket depth reduction when compared with coronally advanced flap alone [27]. In contrast, the 5-year results of a randomized clinical trial using the modified coronally advanced tunnel and CTG to treat single and multiple recessions had drawn that the additional use of Emdogain did not influence the clinical outcomes [28]. In this case report, the Emdogain was used as an adjunct to enhance periodontal regeneration to optimize tissue repair and supporting periodontal healing, but it did not significantly improve the rate of complete root coverage compared to periodontal surgery [29]. Therefore, we thought that the technique can still be performed without Emdogain, although its use may provide additional clinical benefits in terms of soft tissue healing and potential regenerative outcomes.

The limitation of this approach was the requirement of second surgery at the palatal donor site with postoperative patient discomfort. A wide flap can also cause pain for the patient due to increased tissue trauma. Ensuring adequate vascular supply to the grafted tissue is essential for successful integration and healing, but this requires careful handling of the tissues to prevent compromising blood flow [26]. The insertion and fixation of the connective tissue demand precise instrumentation and advanced surgical skills to avoid perforations and ensure the integrity of the overlying tissue. While this present case demonstrated favorable clinical outcomes using the modified rotated flap with a double-component releasing incision and a one-sided tunnel, these findings must be interpreted with caution. As this is a single case report, no direct comparison or claim of superiority over established techniques can be scientifically made. Further studies are required to verify the effectiveness and reliability of this technique.

## 4. Conclusion

The modified rotated flap with a double-component releasing incision and a one-sided tunnel technique is a viable method for managing severe gingival recession following orthodontic treatment. Compared to previously documented methods, this approach improves flap stability while preserving keratinized gingiva. The use of a full-thickness rotational flap is particularly advantageous for thin tissue phenotypes, ensuring an adequate blood supply and minimizing the risk of necrosis. Although additional research and long-term clinical studies are necessary to validate these findings, this technique shows promising potential for achieving optimal root coverage and periodontal health in a single surgical session.

## Data Availability

The data that support the findings of this study are available from the corresponding author upon reasonable request.
